# Causes and Outcomes of Acute Liver Failure in China

**DOI:** 10.1371/journal.pone.0080991

**Published:** 2013-11-22

**Authors:** Pan Zhao, Chunya Wang, Weiwei Liu, Gang Chen, Xinying Liu, Xi Wang, Bao Wang, Liming Yu, Yanrong Sun, Xiaoming Liang, Haozhen Yang, Fei Zhang

**Affiliations:** 1 Liver Failure Therapy and Research Center, Beijing 302 Hospital (PLA 302 Hospital), Beijing, China; 2 Emergency Department, Beijing Anzhen Hospital Affiliated to Capital Medical University, Beijing, China; 3 Postgraduate Division, Academy of Military Medical Science, Beijing, China; 4 Disease Prevention and Control Office, Second Artillery Force of PLA, Beijing, China; 5 Western and Traditional Chinese Medicine Center, Beijing 302 Hospital (PLA 302 Hospital), Beijing, China; 6 Medical Administration Department, Changhai Hospital Affiliated to Second Military Medical University, Shanghai, China; 7 Medical Administration Department, PLA 477 Hospital, Wuhan, Hubei Province, China; 8 Medical Administration Department, General Hospital of Jinan Military Region, Jinan, Shandong Province, China; 9 Medical Administration Department, General Hospital of Lanzhou Military Region, Lanzhou, Gansu Province, China; 10 Gastroenterology Department, PLA 161 Hospital, Wuhan, Hubei Province, China; 11 Intensive Care Unit, General Hospital of PLA, Beijing, China; University of Navarra School of Medicine and Center for Applied Medical Research (CIMA), Spain

## Abstract

**Objectives:**

No extensive investigation has been performed and thus no representative data are available regarding acute liver failure (ALF) in China. This study aims to investigate the causes and outcomes of ALF in China and establish a prognostic model.

**Methods:**

Patients diagnosed as ALF in seven hospitals in different areas of China from January 2007 to December 2012 were retrospectively selected.

**Results:**

Of the 177 patients included in this study, 112 (63.28%) eventually died. The common causes of ALF were drug toxicity (43.50%), indeterminate etiology (29.38%) and acute viral hepatitis (11.30%). Additionally, traditional Chinese herbs predominated in the causes of drug-induced ALF (30/77). No patients in this study received liver transplantation. In the established model for predicting death in ALF, four variables were finally selected out, including age (*P*=0.01), the entry hepatic encephalopathy grade (*P*=0.04), international normalized ratio (*P*<0.01) and arterial blood ammonia (*P*=0.02). Using a threshold value of 0.5683, this model had a sensitivity of 95.24% and a specificity of 91.30%.

**Conclusions:**

Traditional Chinese medicine was a major cause of ALF in China. The spontaneous mortality of ALF was high, whereas the rate of liver transplantation was significantly low. The established prognostic model of ALF had superior sensitivity and specificity.

## Introduction

Acute liver failure (ALF) is a complex condition characterized by rapid deterioration of liver function in a patient without previously recognized liver disease. In spite of the improvement of artificial liver support system and advancement in liver transplantation, the overall mortality of ALF remains 20%-40% [1], the major reasons of which are the rapid disease progression and the highly unpredictable outcomes depending on various factors. Based on previous reports, patients with ALF had greatly variant outcomes according to the causes that also varied markedly by geographical region [2-4]. So, it is important and necessary to do studies on the etiology and prediction for outcome of ALF in different cohorts [5].

Because of its rarity, studies on ALF are relatively fewer, and moreover, discrepancies in the definitions of ALF hinder in-depth worldwide studies. It is reported that more than 40 definitions exit among different countries or regions and the diversity in ALF definitions becomes the obstacle to comparability and quantitative analysis among studies [6]. In China, though liver disease is a major public health concern and viral hepatitis B is especially prevalent [7], the national diagnostic and treatment guidelines for liver failure were issued by Chinese Society of Hepatology in late 2006 [8]. From then on, the diagnosis of ALF began to arrive at uniformity and studies in this field became standardized in China, but the available data on ALF in Chinese population has been still rare up to date. In this study, we performed an extensive investigation including 177 patients diagnosed as ALF after January 2007, with the intentions of investigating the causes and mortality and establishing a prognostic model based on Chinese patients. To the best of our knowledge, this is the first report on a multi-center study of ALF in Chinese population.

## Patients and Methods

### Patient Collection

**Figure 1:**
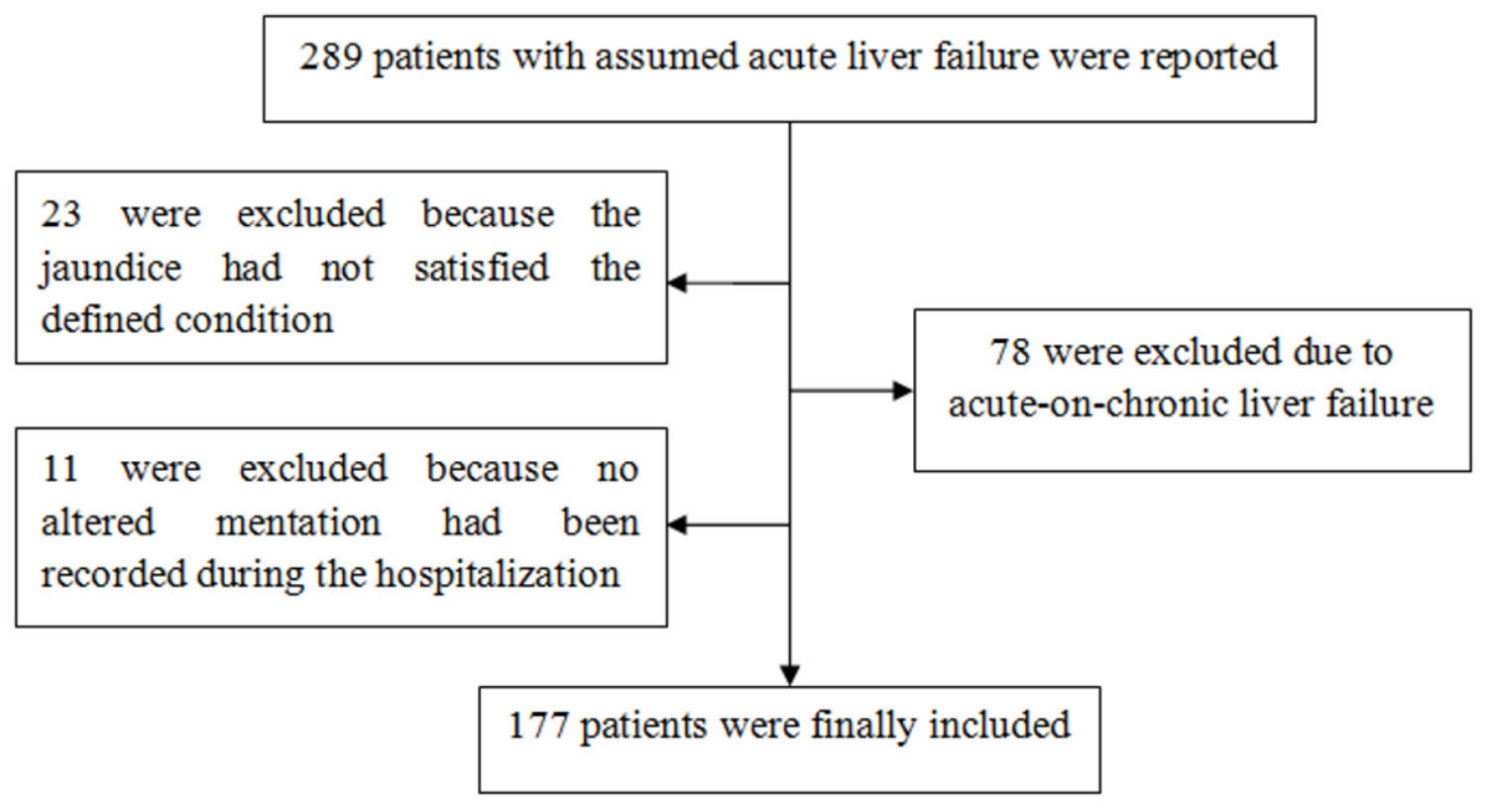
Enrollment of patients in this study.

ALF in this study was defined as coagulopathy (prothrombin activity (PTA)≤40% or international normalized ratio (INR)≥1.5), jaundice (serum total bilirubin (TBil) ≥171 μmol/L or a daily increase ≥17.1 μmol/L) and encephalopathy (any degree of altered mentation) within 4 weeks in a patient without pre-existing liver diseases. Patients older than 12 years eligible for the above diagnostic criteria from January 2007 to December 2012 were included in this study. To ensure the representativeness and comparability of the data, we selected seven tertiary military hospitals in different areas of China: Beijing 302 Hospital (located in Beijing, North China), General Hospital of PLA (located in Beijing, North China), Changhai Hospital Affiliated to Second Military Medical University (located in Shanghai, East China), PLA 161 Hospital (located in Wuhan, Hubei Province, South China), PLA 477 Hospital ((located in Wuhan, Hubei Province, South China), General Hospital of Jinan Military Region (located in Jinan, Shandong Province, East China) and General Hospital of Lanzhou Military Region (located in Lanzhou, Gansu Province, West China). Figure 1 shows the enrollment of patients in our study.

### Ethics Statement

The study was performed in accordance with the ethical guidelines of the 1975 Declaration of Helsinki and was approved by the ethics committees of each hospital. Informed written or verbal consent was obtained from the patient or the next of kin.

### Data Extraction and Assessment

The following variables were obtained from the electronic medical records and follow-up documents: cause, outcome, sex, age, grade of hepatic encephalopathy (HE) on admission, time from onset of illness to the development of HE, white blood cell count, hemoglobin, platelet count, PTA, INR, serum alanine aminotransferase (ALT), serum aspartate aminotransferase (AST), serum alkaline phosphatase (ALP), serum cholinesterase, serum lactate dehydrogenase (LDH), serum TBil, serum albumin, serum creatinine, serum urea nitrogen, serum glucose, serum Na^+^, serum K^+^, serum Cl^-^, arterial blood ammonia (BLA), arterial blood lactate and arterial blood pH. Grade of HE had been determined in the medical records according to the following criteria: I. loss of sleep rhythm, anxiousness, confusion or flapping tremors; II. loss of sphincter control, drowsiness or behavioral disorder; III. persistent coma, but still responding to shouts; IV. deep coma with no consciousness.

### Statistical Analysis

Data analyses were performed using SAS 9.2 software (SAS Institute Inc., Cary, NC, USA). Continuous data were expressed as median (inter-quartile range) or mean±standard deviation. Categorical data were expressed as the number of subjects. Group comparisons were performed using the Wilcoxon rank sum test or t test for continuous variables, and Chi-square test for categorical variables. Logistic regression was used for evaluating prognostic predictors of ALF. Receiver operating characteristic (ROC) curve analysis was used for assessing prognostic accuracy of the outcome prediction model. Tests were two-sided and a probability (*P*) value of less than 0.05 was considered statistically significant.

## Results

### Causes, Mortality and Transplantation

177 patients were totally included in this study. Of these patients, 77 (43.50%) developed ALF caused by drug toxicity, 52 (29.38%) had ALF of indeterminate cause, 20 (11.30%) had acute viral hepatitis and 28 (15.82%) had other causes.

In the patients with ALF induced by drugs, 30 (16.95%) had received herbal remedies, 21 (11.86%) had ingested acetaminophen, 11 (6.21%) had used antibiotics, 5 (2.82%) had received antituberculosis therapy, 5 (2.82%) had received antineoplastic chemotherapy, 2 (1.13%) had used phenprocoumon and 3 (1.69%) had ingested other drugs.

In the patients with ALF due to viral hepatitis, 11 (6.21%) was infected with hepatitis B virus (HBV), 5 (2.82%) with hepatitis E virus (HEV), 2 (1.13%) with hepatitis A virus (HAV), 1 (0.57%) with cytomegalovirus and 1 (0.57%) with Epstein-Barr virus.

Other causes of ALF were respectively ischemic hepatitis in 6 (3.39%) patients, extrahepatic malignancy metastasis in 5 (2.82%), alcoholism in 4 (2.26%), severe infection of biliary tract in 3 (1.69%), poisonous chemical agents in 3 (1.69%), amanita in 3 (1.69%), pregnancy in 2 (1.13%), Budd-Chiari syndrome in 1 (0.57%) and heat stroke in 1 (0.57%).

**Figure 2:**
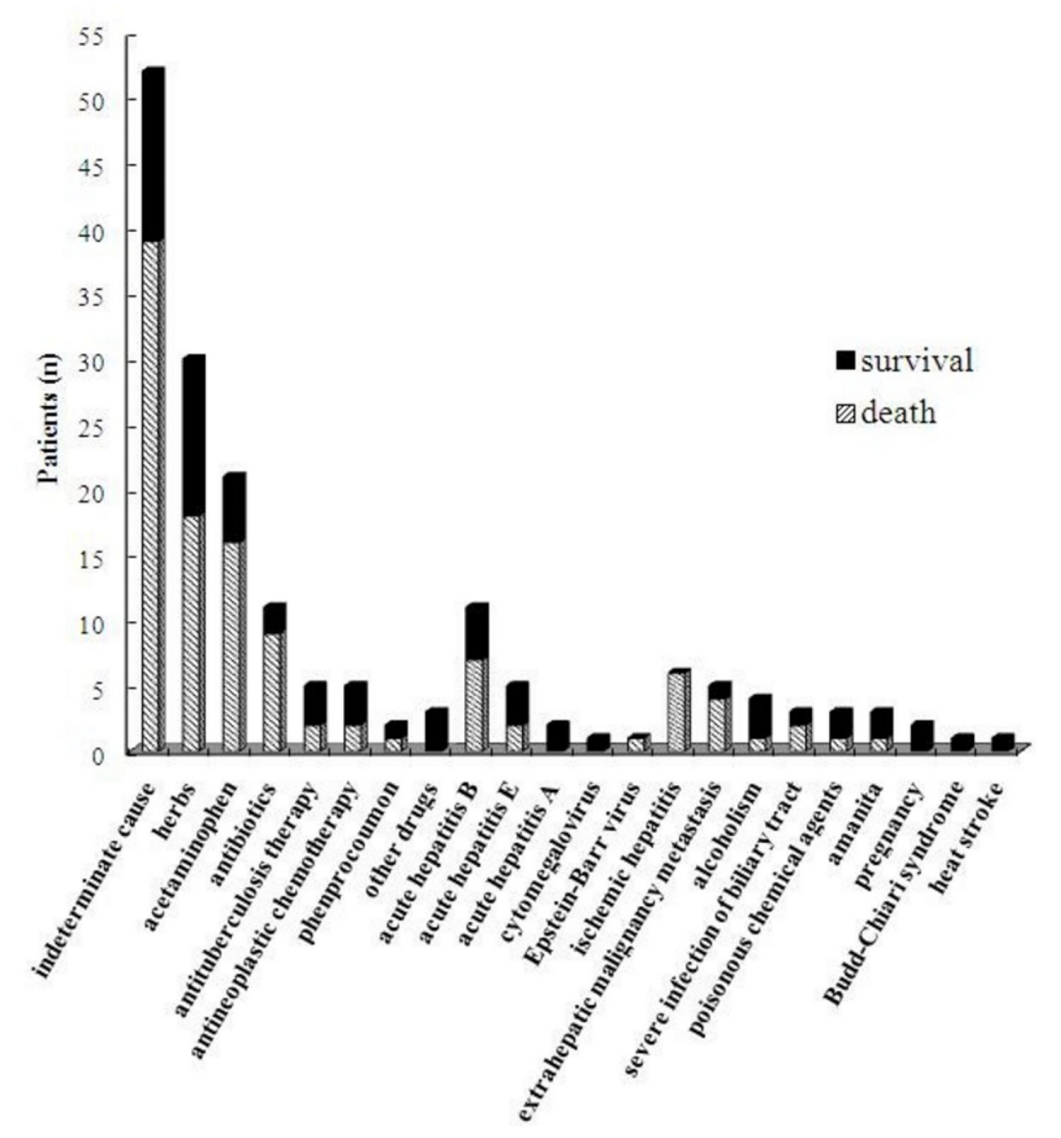
Distribution of causes and outcomes of 177 patients with acute liver failure in China.

No patients received liver transplantation in this study. In the 177 patients, 65 (36.72%) survived and 112 (63.28%) died. The distribution of causes and outcomes of ALF is demonstrated in Figure 2.

### Baseline Characteristics on Admission

Table 1 summarizes the baseline characteristics of patients with ALF on admission according to the outcome (death or survival). The median age (*P*=0.03), INR (*P*<0.01), arterial BLA (*P*<0.01) and serum LDH (*P*<0.01) in patients who died were significantly higher than those in patients who survived, and median platelet count (*P*=0.01) and PTA (*P*<0.01) in patients who died were notably lower than those in patients who survived. In addition, the distribution of HE grade (≤II and II) was statistically different (*P*<0.01). No statistical difference could be detected with respect to other variables.

**Table 1:** Baseline characteristics of patients with acute liver failure on admission and comparison of variables between patients who survived and died.

Parameters	Patients who survived (n=65)	Patients who died (n=112)	*P* value
Sex (male/female)	33/32	49/63	0.37
Age (years)	40.0 (31.0)	46.0 (31.5)	0.03
Etiologies			0.26
Drug (non-herb) toxicity	18	29	
Herbs	12	18	
Indeterminate cause	13	39	
Viral causes	10	10	
Other causes	12	16	
Grade of HE			<0.01
≤II	57	31	
II	8	81	
Days from onset of illness to the development of HE	11 (15)	10 (15)	0.30
Serum ALT (U/L)	796 (1817)	756 (1162)	0.90
Serum AST (U/L)	392.0 (1244.5)	534.0 (824.0)	0.43
Serum Alp (U/L)	162 (83)	165 (87)	0.85
Serum TBil (μmol/L)	326.35±161.32	357.57±152.70	0.76
Serum albumin (g/L)	29 (6)	28 (8)	0.06
Serum cholinesterase (U/L)	2964 (2045)	2895 (1997)	0.33
Serum LDH (U/L)	264.0 (164.0)	366.5 (486.0)	<0.01
Serum creatinine (μmol/L)	84.5 (38.0)	88.0 (57.0)	0.25
Serum urea nitrogen (mmol/L)	4.00 (4.25)	3.9 (5.3)	0.48
Serum glucose (mmol/L)	5.15 (3.13)	5.85 (4.00)	0.06
Serum Na^+^ (mmol/L)	137 (6)	135 (8)	0.08
Serum K^+^ (mmol/L)	3.74±0.59	3.86±0.70	0.29
Serum Cl^-^ (mmol/L)	102.9 (6.9)	100.5 (8.6)	0.14
White blood cell count (×10^9^)	8.35 (5.42)	10.45 (8.61)	0.11
Platelet count (×10^9^)	122 (106)	87 (102)	0.01
Hemoglobin (g/L)	116.5 (34.0)	118.7 (36.5)	0.37
PTA (%)	30.00 (17.21)	16.97 (13.70)	<0.01
INR	1.78 (0.91)	3.39 (1.64)	<0.01
Arterial BLA (μmol/L)	70.50 (28.15)	153.50 (62.00)	<0.01
Arterial blood lactate (mmol/L)	2.75(2.25)	3.40(5.05)	0.42
Arterial blood pH	7.47 (0.08)	7.48 (0.07)	0.63

HE, hepatic encephalopathy; ALT, alanine aminotransferase; AST, aspartate aminotransferase; ALP, alkaline phosphatase; TBil, total bilirubin; LDH, lactate dehydrogenase; PTA, prothrombin activity; INR, international normalized ratio; BLA, blood ammonia.

### Prediction model for the outcome in ALF

**Figure 3:**
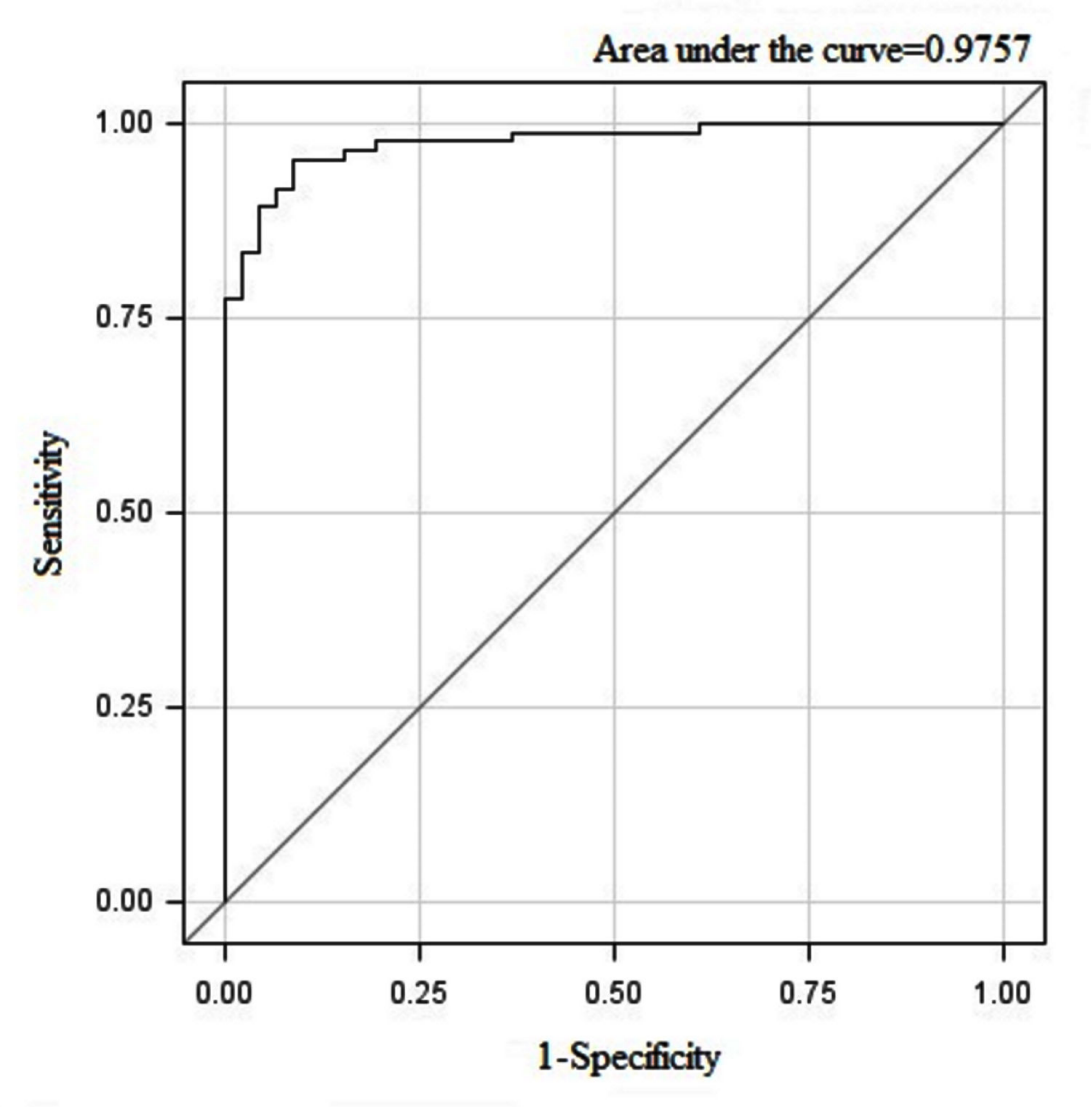
Receiver operating characteristic (ROC) curve analysis for the established prognostic model of acute liver failure.

Table 2 represents the final model established using logistic regression. Four variables were eventually selected out to predict the death of ALF, including age (odds ratio (OR) 1.064, 95% confidence interval (CI) 1.015-1.115, *P*=0.01), the entry HE grade (II *vs.* ≤II, OR 7.459, 95% CI 1.024-54.327, *P*=0.04), INR (OR 10.019, 95% CI 2.530-39.677, *P*<0.01) and arterial BLA (OR 1.035, 95% CI 1.004-1.067, *P*=0.02). Using a threshold value of 0.5683, this model had a sensitivity of 95.24%, a specificity of 91.30% and an accuracy of 93.85%. Figure 3 shows the ROC curve analysis for predictive power of this model (the area under the ROC curve 0.9757, 95% CI 0.9540-0.9973).

**Table 2:** A model for prediction of the death in acute liver failure using entry variables.

Parameters	Odds ratio	95% CI for Odds ratio	*P* value
Age (years)	1.064	1.015-1.115	0.01
Grade of HE (II *vs.* ≤II)	7.459	1.024-54.327	0.04
INR	10.019	2.530-39.677	<0.01
Arterial BLA (μmol/L)	1.035	1.004-1.067	0.02

Somers’D 0.951; Goodman-Kruskal Gamma 0.951; Kendall’s Tau-a 0.438; concordance index 0.976.

HE, hepatic encephalopathy; INR, international normalized ratio; BLA, blood ammonia; CI, confidence interval.

## Discussion

The etiology of ALF showed worldwide variation [9]. Some previous studies reported that synthetic pharmaceuticals (e.g. acetaminophen and phenprocoumon) were the most common cause of ALF in the United States and Europe, while in the Mediterranean region, around 40% of cases with ALF are caused by hepatotropic viruses, most frequently by HBV [2-4]. According to our investigation, the most frequent etiology of ALF in China were drugs, which was in accordance with other reports from western developed countries [2,3,10,11]. However, unlike the overwhelming majority of ALF induced by acetaminophen in western countries, traditional Chinese herbs dominated in the causes in China. So, doctors should pay more attention to the patient’s past medical history on herbal remedy when identifying the etiology of ALF.

Acetaminophen was a well-recognized hepatotoxin [12]. In this study, it was responsible for 11.86% of cases, which was much lower than other reports from the United States and Europe [2,3,10,11]. Unlike the description by Bernal W [13], viral causes of ALF did not predominate in this investigation from China, a developing country, and moreover, acute hepatitis B infection rather than hepatitis E infection, was the main viral cause. In addition to drug-toxicity, indeterminate and viral causes of ALF, 28 (15.82%) cases are classified as other causes, including ischemic hepatitis, extrahepatic malignancy metastasis, alcoholism, etc. Emphatically, ischemic hepatitis was a rare etiology of ALF in China, while it was a common cause of ALF in Sweden [3].

The spontaneous death rates of ALF were reported to range from 10% to 90% in different cohorts [3]. The wide difference was mostly resulted by the discrepancies in the diagnostic criteria of ALF in various studies. In our study, we set coagulopathy, jaundice and encephalopathy as mandatory elements in the definition of ALF. The mortality of ALF without liver transplantation in our study arrived at 63.28%.

Liver transplantation remained the only treatment option in ALF when standard medical therapy failed [14]. In western developed countries, rates of transplant ranged from 25% to 30% in patients with ALF [15], but based on our study, the rate was as low as 0% in China. The reasons for this included the difficulties in obtaining organs in urgent fashion, as well as the economic situation of patients in China. Under such a circumstance, it was critically important to determine the prognosis in ALF as early as possible [16]. With the aim of ensuring the timeliness and practicality of the forecasting model, we used the entry variables rather than the peak ones to predict the outcomes of ALF because it was somewhat late to make further vital decisions when peak value had been reached.

As of now, there have been various prognostic scoring systems applied for predicting outcomes of ALF, including the King’s college hospital criteria, the model for end-stage liver disease score, acute physiology and chronic health evaluation II score and the Clichy criteria [17-19], however, to our knowledge, no prediction model based on Chinese patients with ALF has been established. In this study, the results showed that four common, easily-measured prognostic factors in combination could predict the death in ALF with an accuracy of 93.85%, which were age, the entry HE grade, INR and arterial BLA, and the likelihood of death increased with the levels of the four variables increasing. According to the area under the ROC curve (0.9757), this model also had good discrimination.

The limitation of our research was that we did not set an external validation cohort to verify the established prognostic model.

In conclusion, traditional Chinese medicine was a major cause of ALF in China. The spontaneous mortality of ALF was high, whereas the rate of liver transplantation was significantly low. The established prognostic model of ALF had early applicability and superior sensitivity and specificity.
