# Correction: Causes and Outcomes of Acute Liver Failure in China

**DOI:** 10.1371/journal.pone.0098428

**Published:** 2014-05-16

**Authors:** The PLOS ONE Staff

There are errors in Tables 1 and 2. The “>” symbol was removed while this article was being prepared for publication during the production process. The symbol was also removed in the text “HE grade (≤II and >II)” in the paragraphs preceding each table. Please see the correct tables below.

**Table 1:** Baseline characteristics of patients with acute liver failure on admission and comparison of variables between patients who survived and died.

Parameters	Patients who survived (n = 65)	Patients who died (n = 112)	*P* value
Sex (male/female)	33/32	49/63	0.37
Age (years)	40.0 (31.0)	46.0 (31.5)	0.03
Etiologies			0.26
Drug (non-herb) toxicity	18	29	
Herbs	12	18	
Indeterminate cause	13	39	
Viral causes	10	10	
Other causes	12	16	
Grade of HE			<0.01
≤II	57	31	
>II	8	81	
Days from onset of illness to the development of HE	11 (15)	10 (15)	0.30
Serum ALT (U/L)	796 (1817)	756 (1162)	0.90
Serum AST (U/L)	392.0 (1244.5)	534.0 (824.0)	0.43
Serum ALP (U/L)	162 (83)	165 (87)	0.85
Serum TBil (µmol/L)	326.35±161.32	357.57±152.70	0.76
Serum albumin (g/L)	29 (6)	28 (8)	0.06
Serum cholinesterase (U/L)	2964 (2045)	2895 (1997)	0.33
Serum LDH (U/L)	264.0 (164.0)	366.5 (486.0)	<0.01
Serum creatinine (µmol/L)	84.5 (38.0)	88.0 (57.0)	0.25
Serum urea nitrogen (mmol/L)	4.00 (4.25)	3.9 (5.3)	0.48
Serum glucose (mmol/L)	5.15 (3.13)	5.85 (4.00)	0.06
Serum Na^+^ (mmol/L)	137 (6)	135 (8)	0.08
Serum K^+^ (mmol/L)	3.74±0.59	3.86±0.70	0.29
Serum Cl^-^ (mmol/L)	102.9 (6.9)	100.5 (8.6)	0.14
White blood cell count (×10^9^)	8.35 (5.42)	10.45 (8.61)	0.11
Platelet count (×10^9^)	122 (106)	87 (102)	0.01
Hemoglobin (g/L)	116.5 (34.0)	118.7 (36.5)	0.37
PTA (%)	30.00 (17.21)	16.97 (13.70)	<0.01
INR	1.78 (0.91)	3.39 (1.64)	<0.01
Arterial BLA (µmol/L)	70.50 (28.15)	153.50 (62.00)	<0.01
Arterial blood lactate (mmol/L)	2.75(2.25)	3.40(5.05)	0.42
Arterial blood pH	7.47 (0.08)	7.48 (0.07)	0.63

HE, hepatic encephalopathy; ALT, alanine aminotransferase; AST, aspartate aminotransferase; ALP, alkaline phosphatase; TBil, total bilirubin; LDH, lactate dehydrogenase; PTA, prothrombin activity; INR, international normalized ratio; BLA, blood ammonia.

**Table 2:** A model for prediction of the death in acute liver failure using entry variables.

Parameters	Odds ratio	95% CI for Odds ratio	*P* value
Age (years)	1.064	1.015-1.115	0.01
Grade of HE (>II *vs.* ≤II)	7.459	1.024-54.327	0.04
INR	10.019	2.530-39.677	<0.01
Arterial BLA (µmol/L)	1.035	1.004-1.067	0.02

Somers’D 0.951; Goodman-Kruskal Gamma 0.951; Kendall’s Tau-a 0.438; concordance index 0.976.

HE, hepatic encephalopathy; INR, international normalized ratio; BLA, blood ammonia; CI, confidence interval.

## References

[1] Zhao P, Wang C, Liu W, Chen G, Liu X, et al (2013) Causes and Outcomes of Acute Liver Failure in China. PLoS ONE 8(11): e80991 doi:10.1371/journal.pone.0080991 24278360 10.1371/journal.pone.0080991 PMC3838343

